# Linear-Nonlinear Stiffness Responses of Carbon Fiber-Reinforced Polymer Composite Materials and Structures: A Numerical Study

**DOI:** 10.3390/polym13030344

**Published:** 2021-01-22

**Authors:** S. S. R. Koloor, A. Karimzadeh, M. R. Abdullah, M. Petrů, N. Yidris, S. M. Sapuan, M. N. Tamin

**Affiliations:** 1 Department of Aerospace Engineering, Universiti Putra Malaysia, UPM Serdang 43400, Selangor Darul Ehsan, Malaysia; nyidris@upm.edu.my; 2 Institute for Nanomaterials, Advanced Technologies and Innovation (CXI), Technical University of Liberec (TUL), Studentska 2, 461 17 Liberec, Czech Republic; a.karimzadeh.66@gmail.com (A.K.); michal.petru@tul.cz (M.P.); 3 School of Mechanical Engineering, Universiti Teknologi Malaysia, Johor Bahru 81310, Malaysia; ruslanabdullah@utm.my (M.R.A.); taminmn@fkm.utm.my (M.N.T.); 4 Department of Mechanical Engineering, Universiti Putra Malaysia, UPM Serdang 43400, Selangor Darul Ehsan, Malaysia; sapuan@upm.edu.my

**Keywords:** CFRP composites, material behavior, structural analysis, stiffness response, damage mechanics, finite element method

## Abstract

The stiffness response or load-deformation/displacement behavior is the most important mechanical behavior that frequently being utilized for validation of the mathematical-physical models representing the mechanical behavior of solid objects in numerical method, compared to actual experimental data. This numerical study aims to investigate the linear-nonlinear stiffness behavior of carbon fiber-reinforced polymer (CFRP) composites at material and structural levels, and its dependency to the sets of individual/group elastic and damage model parameters. In this regard, a validated constitutive damage model, elastic-damage properties as reference data, and simulation process, that account for elastic, yielding, and damage evolution, are considered in the finite element model development process. The linear-nonlinear stiffness responses of four cases are examined, including a unidirectional CFRP composite laminate (material level) under tensile load, and also three multidirectional composite structures under flexural loads. The result indicated a direct dependency of the stiffness response at the material level to the elastic properties. However, the stiffness behavior of the composite structures depends both on the structural configuration, geometry, lay-ups as well as the mechanical properties of the CFRP composite. The value of maximum reaction force and displacement of the composite structures, as well as the nonlinear response of the structures are highly dependent not only to the mechanical properties, but also to the geometry and the configuration of the structures.

## 1. Introduction

As a widely used material in advanced industries—such as aerospace, automotive, etc.—fiber-reinforced polymer (FRP) composites have been the subject of many studies [1,2]. Most of these studies have employed a combination of experimental and numerical methods to predict the material or structural behaviors of composites under different loading conditions with respect to their industrial applications [3,4]. Finite element (FE) simulation is the mostly used numerical method because of the availability of well-stablished models and the accuracy of the results [5]. In real-time simulation of structures, the necessary requirement including the precise material properties and constitutive model, as well as correct model configuration, boundary, and loading conditions [1,6,7,8,9]. On the other hand, every FE model and simulation process should be validated through a logically accepted comparison of the FE results with the actual data obtained through experiment or a valid theoretical model [2,4,10,11,12]. 

In many case studies—including material or structures at different micro-macro scales—the validation of FE models has been frequently done by comparing the stiffness curve of solid objects that obtained through FE simulation and compared with their actual behavior measured in the experiments or numerical approach [1,11,13]. This curve is called either stiffness response or load-deformation/displacement, that generally comprise of two parts, that start with an initial linear response to a maximum reaction force at specific displacement, and continued with a nonlinear part to a stage where the load drop is seen [10,14,15]. Some researchers employed the load-displacement curve to validate the numerical simulation of the material behavior [9,16], while others validate the structural response [1,17,18,19]. In this regard, different modes of loading such as tensile, compression, and bending has been investigated [1,17,18,19].

Numerical modelling of the elastic-damage behavior of composite materials and structure is of importance, because the occurrence of invisible damage inside the composite materials and structures such as delamination, fiber breakage, and matrix cracking could cause catastrophic failure of the whole structure [20,21]. Several failure criteria have been derived for damage and failure of FRP composite structures including Chang-Lessard [22] and Greszczuk [23] fiber bucking models, Puck [24] fiber breakage models, Lee [25] fiber failure in both tension and compression, Hashin-Rotem [26] and Shahid-Chang [27] matrix cracking model, Maimí et al. [28] matrix crushing model, etc. In continuum damage mechanics [29,30], the necessity of damage evolution law to be coupled with a suitable failure criteria, in order to estimate the degradation of mechanical properties to fracture is highlighted. Xue and Kirane 2020 [31] and R. Koloor et al., 2018 [1] have developed damage evolution law that enables the prediction of damage initiation and propagation in FRP composites, that are very suitable for estimation of the stiffness response of composite materials and structures. 

Although the theory of composite materials and damage models are used to obtain the stiffness behavior of the structure; however, no studies have been implemented to investigate and describe the stiffness response of the composites in detail and its dependency to the elastic-damage properties at material and structural levels. In this regard, the suitability and applicability of a composite structures to be used for validation of a new damage model or characterization of the mechanical properties through numerical approach, is not determined. On the other hand, the effectiveness of each properties in determination of the mechanical behavior at structural level, has not been studied. Therefore, this study aims to investigate the linear-nonlinear stiffness responses of a carbon fiber-reinforced polymer (CFRP) composites at two levels of material and structure, and describe its dependency to elastic and damage model parameters. For this purpose, four FE models of different composite cases (material and structural levels) are selected for simulation at full damage and failure states, in which the effect of sets of individual and group of elastic and damage parameters are examined. In total, 48 FE models representing the combination of different specimens and properties are run and the results are compared systematically, to provide a comprehensive analysis of stiffness response dependency to the mechanical properties. The results provide important insight into the analysis of composite structures when used for examination of design cases, validation of new theoretical models, and characterization of the mechanical properties.

## 2. Damage Model of FRP Composite Material 

In modelling aspect, each lamina is treated as a homogeneous orthotropic layer. Classical lamina theory is used to model composite lamina [32]. To describe the material properties and constitutive model of the unidirectional (UD) lamina, two coordinate systems are required, the material (1-, 2-, 3-axis) and global (x-, y-, z-axis) coordinates. In the material coordinate system, axis 1 and 2 are in the plan of lamina, where axis 1 is along the fibers and axis 2 is normal to the fibers. Therefore, the stress in an equivalent thin lamina (i.e., material coordinate), which is under plane stress condition, can be written in terms of the global strain as [17]

(1) $$\begin{array}{c} \underset{\left\{\hat{\sigma }\right\}}{\underbrace{\left\{\begin{array}{c} \sigma_{11}\\ \sigma_{22}\\ \tau_{12} \end{array}\right\}}}=\underset{\left[T\right]}{\underbrace{\left[\begin{array}{ccc} \cos^{2}\theta & \sin^{2}\theta & 2\sin\theta \cos\theta \\ \sin^{2}\theta & \cos^{2}\theta & -2\sin\theta \cos\theta \\ -\sin\theta \cos\theta & \sin\theta \cos\theta & \cos^{2}\theta -\sin^{2}\theta \end{array}\right]}}\\ \times \underset{\left[{\bar{Q}}_{ij}\right]}{\underbrace{\left[\begin{array}{ccc} {\bar{Q}}_{11} & {\bar{Q}}_{12} & {\bar{Q}}_{16}\\ {\bar{Q}}_{12} & {\bar{Q}}_{22} & {\bar{Q}}_{26}\\ {\bar{Q}}_{16} & {\bar{Q}}_{26} & {\bar{Q}}_{66} \end{array}\right]}}\times \underset{\left\{\varepsilon \right\}}{\underbrace{\left\{\begin{array}{c} \varepsilon_{x}\\ \varepsilon_{y}\\ \gamma_{xy} \end{array}\right\}}} \end{array}$$

where {σ} is the material (local) stress tensor, [T] is the axis rotation matrix, $\left[{\bar{Q}}_{ij}\right]$ is the stiffness matrix which determines the mechanical properties of the lamina, {ε} is the strain tensor in the global coordinate, and θ is the lamina angle. In this equation, mainly the elastic properties that are obtained through standard tests [33], are used to compute the linear behavior of the composite laminate [17,34].

There are several constitutive damage models for defining the FRP composites failure [3,4,35,36,37,38]. A constitutive damage model of lamina [1] is applied for the simulation of elastic-damage behavior of the FRP composite materials and structures. In this model, each damage mode is defined by a bilinear curve that represents the elastic behavior and the softening process in each mode of loading (refer to Section 3). 

### 2.1. Damage Initiation

The initiation of damage is predicted by the Hashin’s failure model in laminas at meso-scale [35]. The stress-based Hashin’s criteria are described for fiber and matrix phases in the lamina under plane stress condition, as follows:

For the fiber fracture, buckling or kinking:

(2a) $${\left(\frac{{\hat{\sigma }}_{11}}{X^{T}}\right)}^{2}+{\left(\frac{{\hat{\tau }}_{12}}{S^{L}}\right)}^{2}=d_{f}^{t};\;\mathrm{if}\;{\hat{\sigma }}_{11}\geq 0\;\left(\mathrm{Tension}\right)$$

(2b) $${\left(\frac{{\hat{\sigma }}_{11}}{X^{C}}\right)}^{2}=d_{f}^{c};\;\mathrm{if}\;{\hat{\sigma }}_{11}<0\;\left(\mathrm{Compression}\right)$$

For the matrix cracking and crushing:

(2c) $${\left(\frac{{\hat{\sigma }}_{22}}{Y^{T}}\right)}^{2}+{\left(\frac{{\hat{\tau }}_{12}}{S^{L}}\right)}^{2}=d_{m}^{t};\;\mathrm{if}\;{\hat{\sigma }}_{22}\geq 0\left(\mathrm{Tension}\right)$$

(2d) $${\left(\frac{{\hat{\sigma }}_{22}}{2S^{T}}\right)}^{2}+\left[{\left(\frac{Y^{C}}{2S^{T}}\right)}^{2}-1\right]\left(\frac{{\hat{\sigma }}_{22}}{Y^{C}}\right)+{\left(\frac{{\hat{\tau }}_{12}}{S^{L}}\right)}^{2}=d_{m}^{c};\;\mathrm{if}\;{\hat{\sigma }}_{22}<0\;\left(\mathrm{Compression}\right)$$

In these equations, ${\hat{\sigma }}_{ij}$ are the effective stresses in the meso-scale lamina, *X^T^, Y^T^, X^C^, Y^C^, S^L^*, and *S^T^* are the strength properties, and the parameters $d_{f}^{t}$, $d_{f}^{c}$ and $d_{m}^{t}$, $d_{m}^{c}$ shows the internal damage variables, in which the index represents the fiber (f) and matrix (m) phases, and the power expresses tensile (t) and compression (c) loading modes. The strength properties are normally extracted through standard test method such as tension, compression, and shear experiment on 0o and 90o standard samples [33].

### 2.2. Post-Damage Initiation

The orthotropic intrinsic of the FRP Composite lamina causes the occurrence of mixed-mode damage and multiple failures [17,34]. Once damage initiated, this effect could be applied by updating the elastic stress tensor shown in Equation (1), by multiplying by a damage variable matrix as

(3a) $${\hat{\sigma }}_{ij}=\;\{\begin{array}{c} \sigma_{ij},\;before\;damage\;initiation\\ D\sigma_{ij},\;If\;any\;damage\;initiated \end{array}$$

where ${\hat{\sigma }}_{ij}$ is the effective stress and *D* is the damage variable matrix, which obtained from the strain equivalence hypothesis, as [7,29,34,37,39]

(3b) $$D=\left[\begin{array}{ccc} 1/\left(1-d_{f}\right) & 0 & 0\\ 0 & 1/\left(1-d_{m}\right) & 0\\ 0 & 0 & 1/\left(1-d_{s}\right) \end{array}\right]$$

in which $d_{f},\;d_{m}$ and $d_{s}$ derive from the internal damage variables in the lamina (i.e., Equations (2a)–(2d))

(3c) $$d_{m}=\{\begin{array}{c} d_{m}^{t}\;\mathrm{if}\;{\hat{\sigma }}_{22}\geq 0,\\ d_{m}^{c}\;\mathrm{if}\;{\hat{\sigma }}_{22}<0, \end{array}\;d_{f}=\{\begin{array}{c} d_{f}^{t}\;\mathrm{if}\;{\hat{\sigma }}_{11}\geq 0,\\ d_{f}^{c}\;\mathrm{if}\;{\hat{\sigma }}_{11}<0, \end{array}\;d_{s}=1-\left(1-d_{m}^{t}\right)\left(1-d_{m}^{c}\right)\left(1-d_{f}^{t}\right)\left(1-d_{f}^{c}\right)$$

The effective stress parameters are applied in Hashin model to predict the initiation of damage at different levels. 

### 2.3. Damage Propagation

The propagation of damage in the FRP composite lamina is modeled by applying energy-based criteria to define the softening behavior to the final failure [34]. Based on these criteria, during the damage evolution, the relation between the equivalent stress and displacement in each failure mode is expressed by [34]:

Fiber tension $\left({\hat{\sigma }}_{11}\geq 0\right)$:

(4a) $$\sigma_{eq.}=\left(\frac{{\left(\langle \sigma_{11}^{o}\rangle \langle \varepsilon_{11}^{o}\rangle +\tau_{12}^{o}\varepsilon_{12}^{o}\right)}^{2}}{\left(\left(L^{c}\left(\langle \sigma_{11}^{o}\rangle \langle \varepsilon_{11}^{o}\rangle +\tau_{12}^{o}\varepsilon_{12}^{o}\right)\right)-2G_{C}^{XT}\right)\times \left(\langle \varepsilon_{11}^{o}\rangle {}^{2}+\varepsilon_{12}^{o}{}^{2}\right)}\right)\times \left(\delta_{eq.}-\frac{2G_{C}^{XT}\;\sqrt{\langle \varepsilon_{11}^{o}\rangle {}^{2}+\varepsilon_{12}^{o}{}^{2}}}{\;\langle \sigma_{11}^{o}\rangle \langle \varepsilon_{11}^{o}\rangle +\tau_{12}^{o}\varepsilon_{12}^{o}}\right)$$

Fiber compression $\left({\hat{\sigma }}_{11}<0\right)$:

(4b) $$\sigma_{eq.}=\left(\frac{\langle -\sigma_{11}^{o}\rangle {}^{2}}{\left(L^{c}\langle -\varepsilon_{11}^{o}\rangle \langle -\sigma_{11}^{o}\rangle -2G_{C}^{XC}\right)}\right)\times \left(\delta_{eq.}-\frac{2G_{C}^{XC}\;}{\langle -\sigma_{11}^{o}\rangle }\right)$$

Matrix tension $\left({\hat{\sigma }}_{22}\geq 0\right)$:

(4c) $$\sigma_{eq.}=\left(\frac{{\left(\langle \sigma_{22}^{o}\rangle \langle \varepsilon_{22}^{o}\rangle +\tau_{12}^{o}\varepsilon_{12}^{o}\right)}^{2}}{\left(\left(L^{c}\left(\langle \sigma_{22}^{o}\rangle \langle \varepsilon_{22}^{o}\rangle +\tau_{12}^{o}\varepsilon_{12}^{o}\right)\right)-2G_{C}^{YT}\right)\times \left(\langle \varepsilon_{22}^{o}\rangle {}^{2}+\varepsilon_{12}^{o}{}^{2}\right)}\right)\times \left(\delta_{eq.}-\frac{2G_{C}^{YT}\;\sqrt{\langle \varepsilon_{22}^{o}\rangle {}^{2}+\varepsilon_{12}^{o}{}^{2}}}{\langle \sigma_{22}^{o}\rangle \langle \varepsilon_{22}^{o}\rangle +\tau_{12}^{o}\varepsilon_{12}^{o}}\right)$$

Matrix compression $\left({\hat{\sigma }}_{22}<0\right)$:

(4d) $$\sigma_{eq.}=\left(\frac{{\left(\langle -\sigma_{22}^{o}\rangle \langle -\varepsilon_{22}^{o}\rangle +\tau_{12}^{o}\varepsilon_{12}^{o}\right)}^{2}}{\left(L^{c}\left(\langle -\sigma_{22}^{o}\rangle \langle -\varepsilon_{22}^{o}\rangle +\tau_{12}^{o}\varepsilon_{12}^{o}\right)-2G_{C}^{YC}\;\right)\left(\langle -\varepsilon_{22}^{o}\rangle {}^{2}+\varepsilon_{12}^{o}{}^{2}\right)}\right)\times \left(\delta_{eq.}-\frac{2G_{C}^{YC}\;\sqrt{\langle -\varepsilon_{22}^{o}\rangle {}^{2}+\varepsilon_{12}^{o}{}^{2}}\;}{\langle -\sigma_{22}^{o}\rangle \langle -\varepsilon_{22}^{o}\rangle +\tau_{12}^{o}\varepsilon_{12}^{o}}\right)$$

where $L^{c}$ is the length of a first-order element on the lamina, and this form of brackets, <•>, represents the Macaulay bracket. In these equations $G_{C}^{XT}$, $G_{C}^{XC}$, $G_{C}^{YT}$, and $G_{C}^{YC}$ are the values of fracture energy in fiber and transvers-to-fiber directions for each mode of loadings, in which has been obtained through standard [33] and innovative [40] experimentations. The parameter $\sigma_{ij}^{o}$, $\tau_{ij}^{o}$, and $\varepsilon_{ij}^{o}$ are the effective stresses, which computed at the damage initiation using the following damage evolution parameter [34,41]

(5) $$d=\frac{\delta_{\mathrm{eq}}^{f}\left(\delta_{\mathrm{eq}}-\delta_{\mathrm{eq}}^{0}\right)}{\delta_{\mathrm{eq}}\left(\delta_{\mathrm{eq}}^{f}-\delta_{\mathrm{eq}}^{0}\right)}\;\delta_{\mathrm{eq}}\geq \delta_{\mathrm{eq}}^{0}$$

where $\delta_{\mathrm{eq}}^{0}$ represents the equivalent displacement at the onset of damage (i.e., d = 0), and $\delta_{\mathrm{eq}}^{f}$ is the equivalent displacement at failure of a material point (i.e., d = 1). Further explanation about these criteria for each level of elastic-damage behavior of the FRP composite, i.e., damage initiation, post damage initiation, and damage propagation, is found in [1,17,34].

## 3. Mechanical Properties and Damage Model Parameters

**Figure 1:**
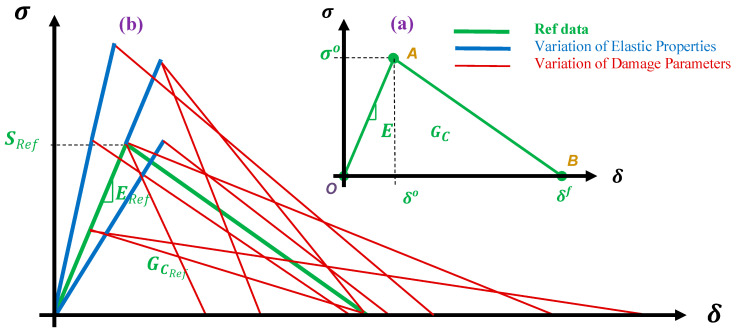
Bilinear damage model of FRP composites (**a**) as described by the theory, and (**b**) the variation of this model based on different mechanical and damage properties reported in the previous studies.

The mathematical formulation of the constitutive damage model describing the elastic to damage and fracture of FRP composites, is described in Section 2. The schematic bilinear curve representing the constitutive model is shown in Figure 1a. This constitutive curve includes a linear section (line ***OA***) to model the elastic behavior, which depends to nine properties of elastic moduli, shear moduli, and Poisson’s ratio (Equation (1)) [2,32]. The maximum elastic behavior is concluded in the yielding point ($\delta^{o}$*,*$\sigma^{o}$) which represents the onset of damage and depends to the six strength properties of matrix and fiber in tension and compression loading conditions (Equations (2a)–(2d)) [35,42,43]. Then, a second linear line (***AB***) representing the damage evolution process to fracture ($\delta^{f}$) that is calculated based on fracture energy value in each different failure modes (Equations (4a)–(4d)) [1,34,44,45]. These mechanical properties are essential to compute a full elastic-to-failure process at material point, that are obtained through standard test processes [33]. In the past three decades, many studies (e.g., [46,47,48,49,50,51,52,53]) have used these mechanical parameters to investigate the behavior of composite parts, in which a range of different value with 50–200% tolerance have been reported. Here, a schematic image of the average values of those works in the form of different set of parameters are present in Figure 1b. In this figure, the central data shown using green-line is the reference data, which are validated and presented by the same author in the previous works [1,17,44,45], as listed in Table 1. It is worth mentioning that Figure 1b provides an general view of the scattered data (elastic and damage parameters), and highlights the range of the considered values in the view of a representation of the constitutive damage model. It should be noted that the authors are aware of the fact that, these values may change for different carbon fibers or thermoset matrix constituents that are used in the manufacturing of CFRP composite structures. However, the objective is to show the variations of mechanical parameters in view of its effect in the constitutive model. 

**Figure 2:**
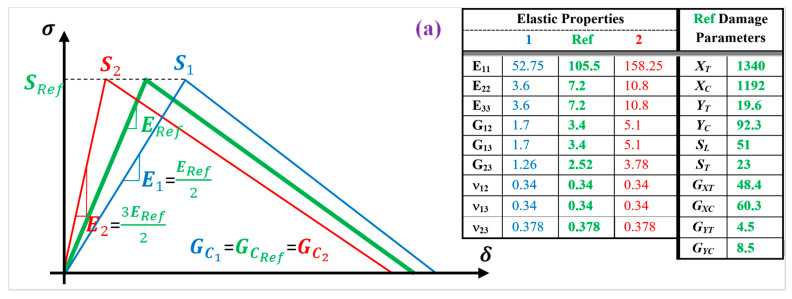

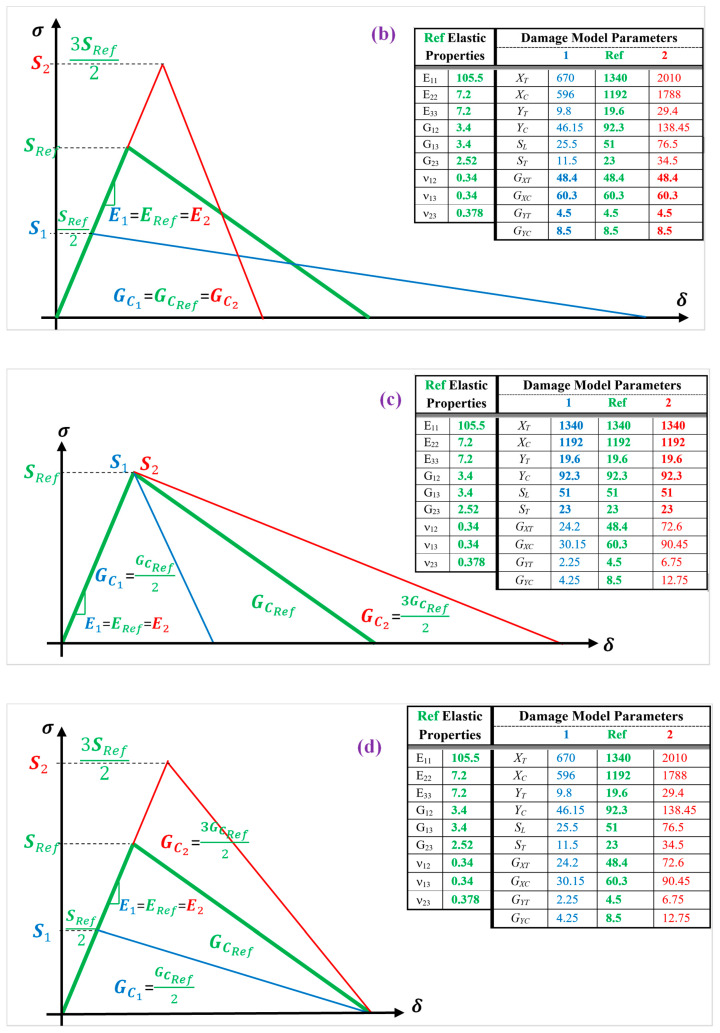
Bilinear damage model of FRP composites based on different (**a**) elastic, (**b**) strength, (**c**) fracture energy, and (**d**) strength and energy properties.

As mentioned, the main mechanical properties and damage model parameters that are used as the ref data (green-color curve, Figure 1) for the simulation of CFRP composite materials and structures, are extracted from the published work of the same authors [1,17,44,45], which are rigorously examined and validated through mechanical analysis of different structures. These properties are listed in Table 1. To define a set of elastic and damage properties to build a systematic study and investigate the effect of mechanical properties on the stiffness response of the composite materials and structures, two different ranges of properties (along the reference properties) one 50% of the ref data (one-half ref) and second 1.5 times of the ref properties (three-half ref) are selected. It is important to make a note that, mechanical properties similar to the range of one-half ref and three-half ref properties, have been reported as the properties of CFRP composite in the previous studies. These sets of different properties projected from different values reported in the previous studies (e.g., [46,47,48,49,50,51,52,53] as shown in Figure 1b), are used for the parametric study on the stiffness responses of composite materials and structures (Figure 1), as listed in Table 1. The detail information about the property combinations is provided in Figure 2, using both tables and schematic view of the bilinear damage model curves. In all cases, a full damage model comprising elastic, strength, and energy properties are used. The CFRP composite properties used in this investigation are considered from a real structure that has been manufactured using pre-impregnated laminas (prepreg) with high modulus carbon fibers with epoxy resin (M40J fibers and NCHM 6376 resin, Structil France) [1,17], in order to make a realistic numerical study.

In the first set of properties (Figure 2a), the variation of the elastic properties on the stiffness response of the composite material and structures, is examined while the strength and energy properties selected from the Ref data. In the second set of properties (Figure 2b), the variation of the strength properties is examined on the stiffness response, while the elastic and energy properties are assumed similar to the ref data. In the third set of properties (Figure 2c), the variation of the energy properties is examined, while the elastic and strength properties are defined based on the ref data. Finally, in the fourth set of properties (Figure 2d), the variation of the strength and energy properties are examined on the stiffness response of the composite cases, while the elastic properties are assumed similar to the ref data.

## 4. Finite Element Model and Simulation Process 

**Figure 3:**
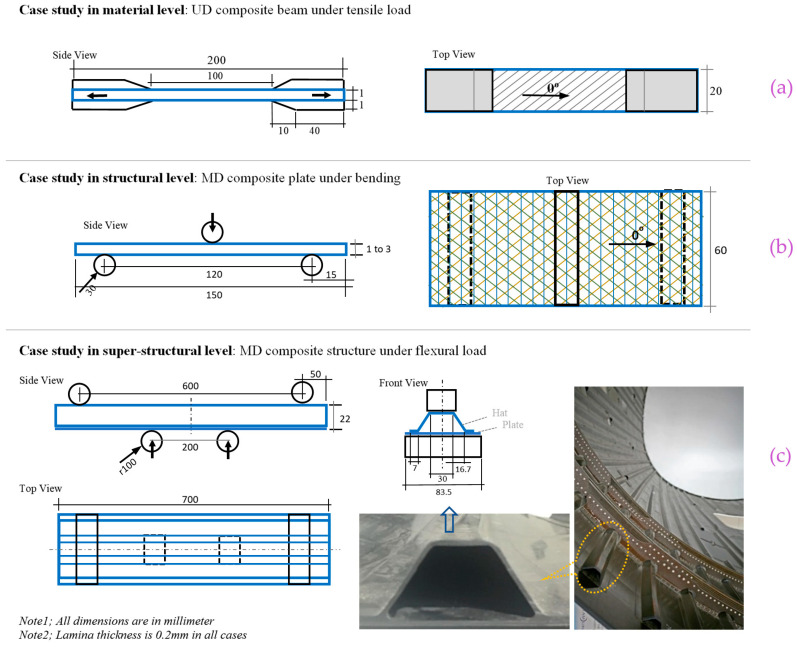
Specimens configuration at different levels of material level (**a**), structural level (**b**), and super-structural level (**c**) as an example of hat-stiffened composite section in the Fuselage section of Boeing 787.

In this study, the stiffness response of CFRP composite material and structures are investigated with respect to the variation of the elastic-damage properties. Composites are normally tested at different levels of material, structure, or super-structure. A list of specimens at different material and structural levels that are considered in the computing activities of the present study, are provided in Table 2, and their configuration and geometry are shown in Figure 3. 

The material level tests such as UD 0° or 90° FRP composite under tensile or compressive loadings [33], are normally used for the extraction of mechanical properties, which are not a good target for this study. However, an angle laminate made of several UD lamina with an arbitrary angle which is the same in all layers, is a good case for the simulation of composite at material level. Because, the response of the material is reflected by a combination of normal and shear stresses and deformations. In this regard, a beam made of five layers of 45° UD lamina is considered for data analysis at materials level, as shown in Figure 3a. The stiffness response at structural level is investigated by using a MD composite plate under bending, as shown in Figure 3b. Two cases are considered with different fiber arrangements and thicknesses, to highlight the structural damage growth dependency to the mechanical properties. A second stage of the investigation is implemented by modelling of a super-structure under flexural loading condition. In this regard, a hat-stiffened part of composite structures that are used in aerospace application, is modelled under four-point bending, as shown in Figure 3c. The flexural load applied from the bottom part of the structure is to apply the load in a more convenient way, because in the real application, the loading will not appear on the hat section.

The four different sets of properties shown in Figure 2, are used in four models of the composite at the material and structural levels described in Figure 3. As the result of that, 48 FE models are developed, which are listed and coded in Table 3. The case codes are used to name the results of stiffness response for brevity and referment to each case.

### 4.1. Finite Element Simulation 

**Figure 4:**
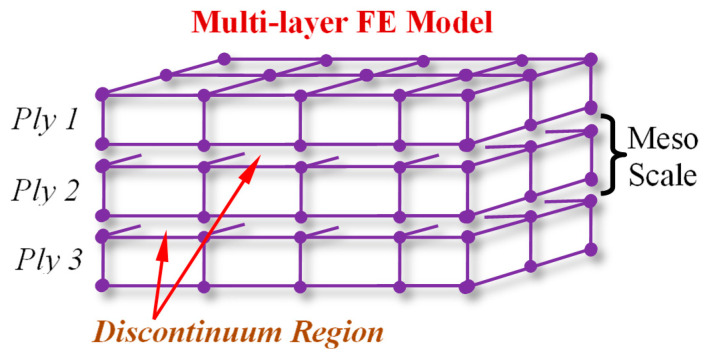
FE model of multi-layer construction of laminated composite.

In this study, CFRP laminated composite materials and structures are simulated using a multilayer construction FE model. In this model each lamina is modelled as a homogeneous orthotropic layer, that created using 8-node three-dimensional continuum shell elements in FE environment. All laminas are stacked with their specific orientation on each other, in which a weak physical interface is considered between the adjacent layers, as shown in Figure 4. This FE model that has been validated in the previous study [1] for the CFRP laminated composite with the reference constitutive model, is applied to the simulation of the material and the structures described in Figure 3. The multilayer structure is more suitable for the simulation of the progressive damage behavior of the CFRP composite laminate manufactures using the autoclave or prepreg method, in which the prepreg laminas are chemically attached together in the curing procedure. The detailed descriptions about the model creation, simulation process, mesh convergence study, and the effect of the intralayer interface can be found in [17,34].

**Figure 5:**
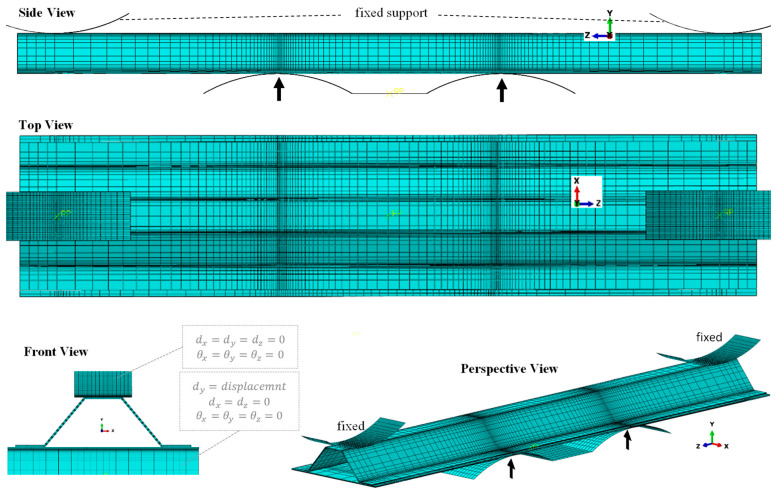
Mesh configuration of the 3D geometry and boundary condition of the sample.

The geometry of the materials and structural models were created based on the dimensions of the specimens listed in Table 2. As an example, the 3D geometry, load, and boundary condition of the composite structure (Profile, Table 2) with the mesh configuration are demonstrated in Figure 5. The specimens were meshed using SC8R elements [37], in which the average element size is 0.2 mm in the damage areas. For the loadings tools and supports a frictionless contact with the specimen is considered in which rigid body element with R3D4 types are applied [37]. In this simulation the interfaces between the plies are perfectly bonded, in which zero relative motion is considered. To eliminate the effect of mesh size on the results of the simulations, mesh convergence study is done in two levels; firstly, the optimum element size is investigated for the pure elastic simulation, and then the final element size is found for the elastic-damage model, in which the detail is provided elsewhere [17]. The mesh configurations of the other cases are created with the same procedure according to the geometry shown in Figure 3, in which example of them could be find previous published work [1,45]. 

### 4.2. Validation of the Finite Element Model 

Numerical method has become one of the important tools for design and analysis of composite structures. An accurate numerical analysis of FRP composite materials and structures, very much depend on the credibility of the input-data, material model, and simulation processes. The material properties used in this study, has been measured experimentally and examined thorough several cases of multidirectional CFRP composite structure under tensile, bending and flexural loading, as reported in the published literature [33,34]. The material model, and FE simulation process are also used in model creation of several CFRP composite cases which have been validated experimentally [1,8,17,44,45,54,55]. In this study, the same material properties, and simulation processes have been utilized to create three distinct composite cases at different levels, to highlight the dependency of the mechanical properties to the global stiffness response.

## 5. Results and Discussion 

The FE results of the material and structural stiffness in term of global load–displacement curve are presented quantitatively and discussed in this section. Results are divided into two main parts, firstly the influences of elastic properties are shown, and secondly the effect of damage parameters will be discussed. 

### 5.1. Stiffness Response with Respect to the Variation of Elastic Properties

**Figure 6:**
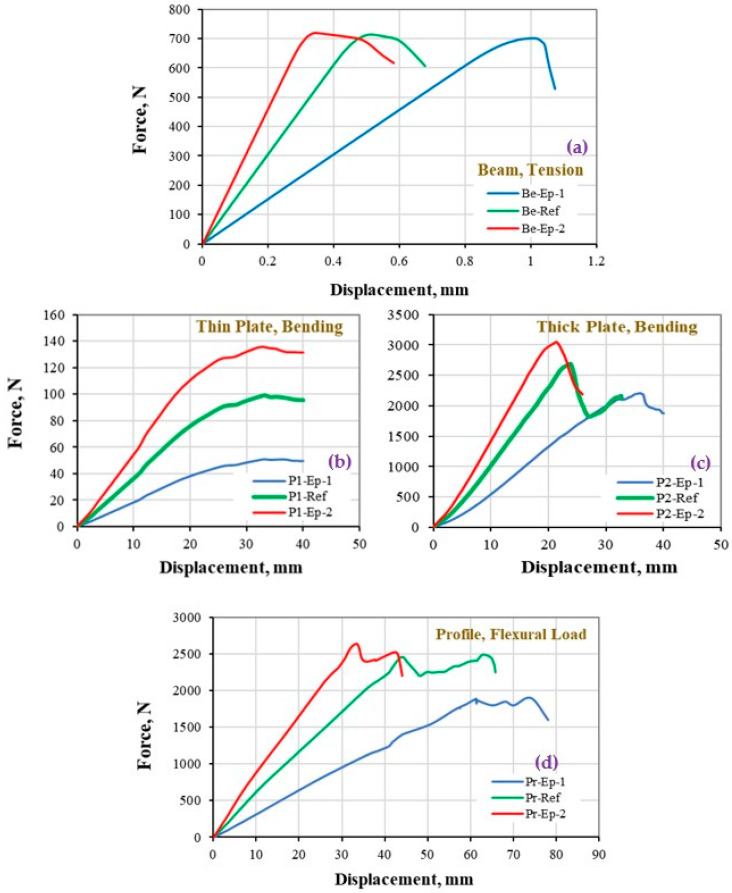
Stiffness response of CFRP composites at (**a**) material, (**b**,**c**) structure, and (**d**) super-structure levels, to the variation of the elastic properties.

The load-displacement/deflection responses of the CFRP composite under tensile and flexural loads are presented in Figure 6. The legend provided in each picture has been given in Table 3. The reference curve that has been validated by the experiment is shown using green color, while the results obtained from one-half and third-half elastic properties are shown using blue and red color curves, respectively. The effect of elastic properties appears significant in changing the stiffness response of the structures especially in the linear region. Since the composite laminate is selected with 45° laminas angle specification, both elastic and shear moduli affect the slope of the global load-displacement response. However, the values of shear moduli (1.2–5.1 MPa) are almost 3–30 time less than elastic moduli values (3.6–158 MPa), as given in Table 1, which have led to the disproportional slope in one-half and third-half responses compare to the ref data. 

Considering that in this section, the focus is only on the variation of elastic properties while the damage properties are kept the same in all cases, it is worth to highlight that while the maximum reaction force of the specimens are computed similarly (≈700 N) at the material level (Figure 6a), it has been changed significantly at the structural level (Figure 6b–d). In addition, it should be noted that, the deformation at maximum load is also affected to be larger for thick plates (Figure 6c) and profile cases (Figure 6d). Such effects were seen as the response to geometry changes in the structure cases that is intensified for the structures with larger geometry.

### 5.2. Stiffness Response with Respect to Variation of Damage Parameters

In this section, the stiffness responses of CFRP composite at material and structural levels are investigated with respect to the variation of the damage properties (strength and fracture energy), while the elastic properties are kept similar to the ref data. The FE result of each specimen is provided separately and discussed. The results of each specimen are shown using three graphs that illustrate first the effect of strength variation, second the effect of fracture energy variations, and third the effect of both strength and energy variations, that provides a systematic understanding about the effect of the damage parameters in detail. The reference response is shown using green color curve, while one-half damage properties and three-half properties are shown using blue and red color curves, respectively. The code of cases provided in the legend, has been given in Table 3. The values of horizontal and vertical axes are unified for all cases, to show a consistent comparison between the results.

#### 5.2.1. CFRP Composite Beam under Tension

**Figure 7:**
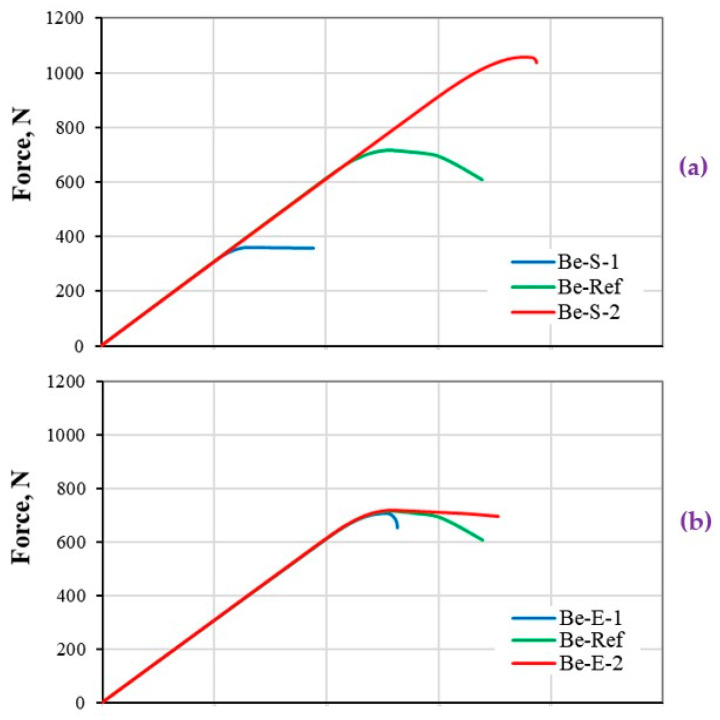

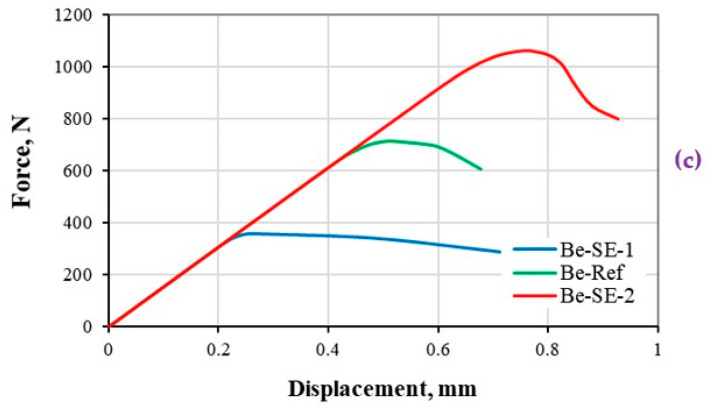
Stiffness response of CFRP composites beam (material level) under tensile load. The load–displacement response based on (**a**) strength, (**b**) energy, and (**c**) both strength and energy properties variations.

**Figure 8:**
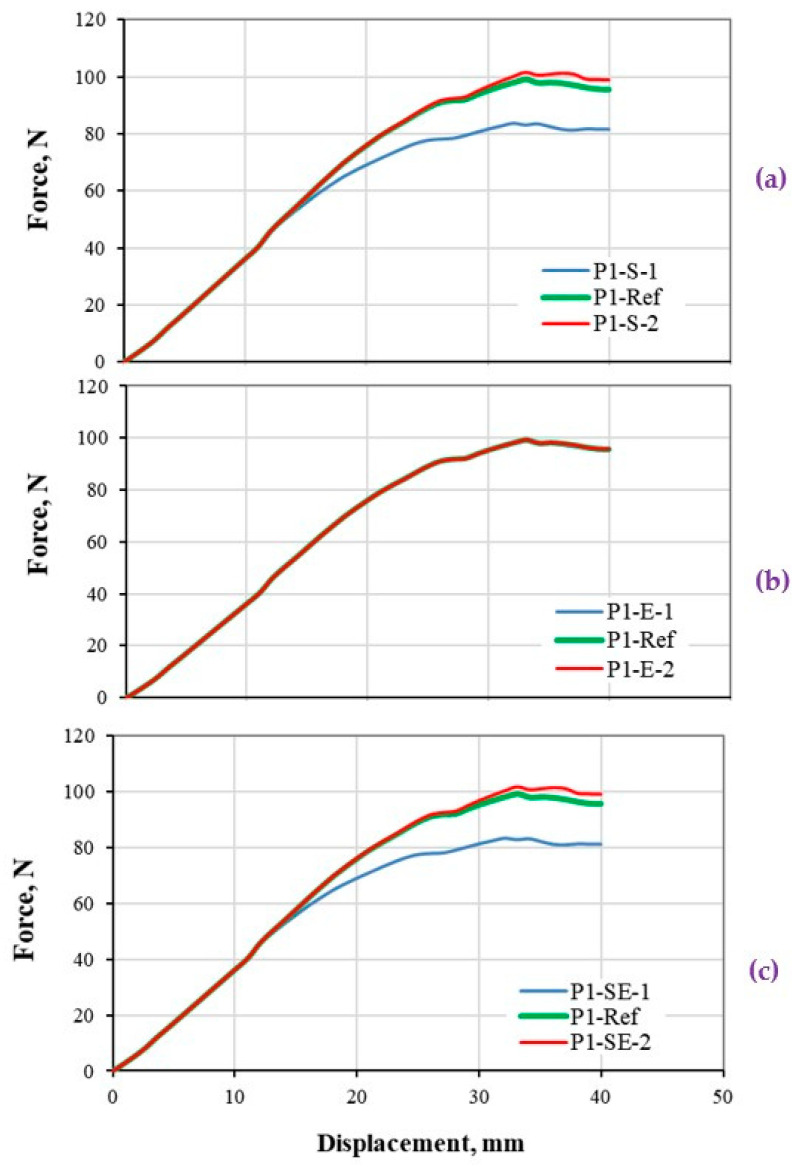
Stiffness response of thin CFRP composites plate (structural level) under three-point bending load. The load-displacement response based on (**a**) strength, (**b**) energy, and (**c**) both strength and energy properties variations.

**Figure 9:**
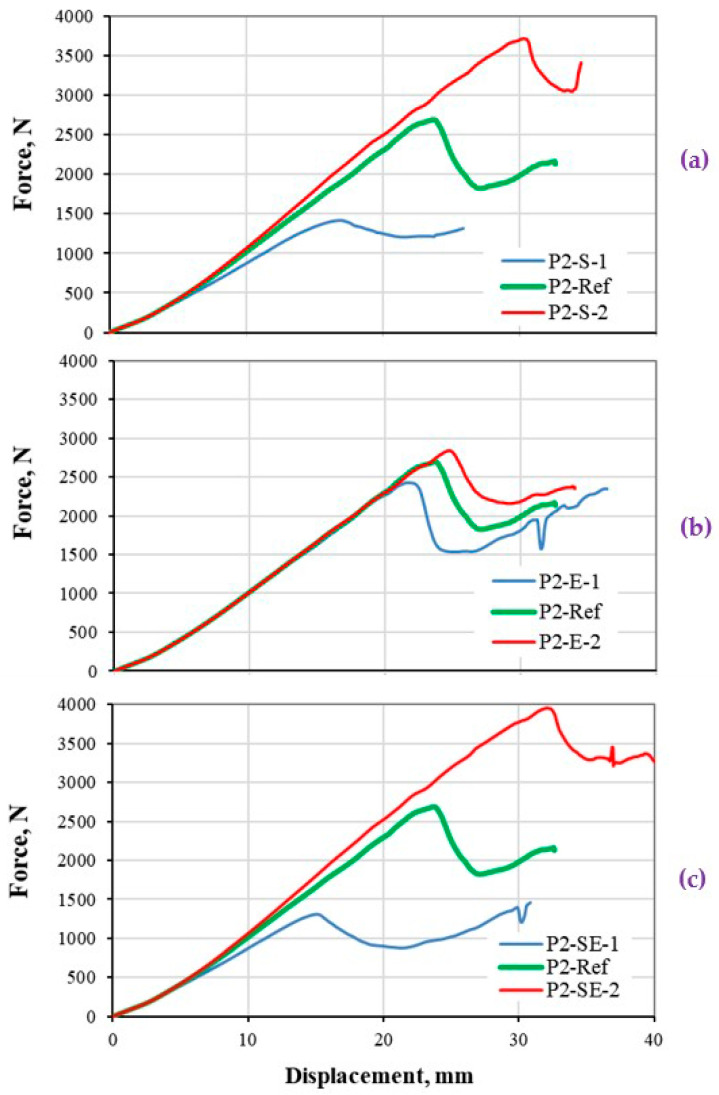
Stiffness response of thick CFRP composites plate (structural level) under three-point bending load. The load-displacement response based on (**a**) strength, (**b**) energy, and (**c**) both strength and energy properties variations.

**Figure 10:**
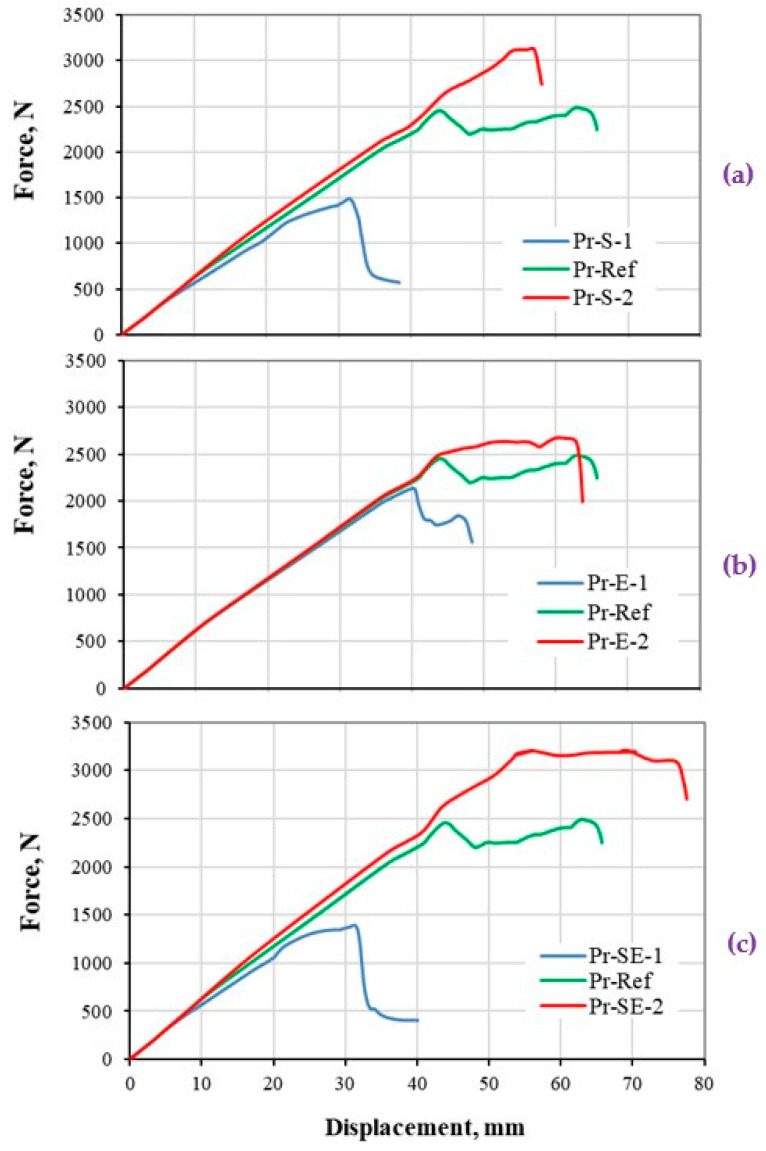
Stiffness response of hat-stiffened structure made of CFRP composites (super-structural level) under four-point bending load. The load-displacement response based on (**a**) strength, (**b**) energy, and (**c**) both strength and energy properties variations.

The load-displacement responses of the CFRP composite beam under tensile load are presented in Figure 7. Although the results seem to be simply understandable, however it will help to comprehend the variation of stiffness response in complex condition at structural level shown in Figure 8, Figure 9 and Figure 10.

Figure 7a indicates a direct effect on the maximum reaction force of the composite beam based-on the variation of strength property. Considering a bigger or smaller strength value, causes the bilinear curve to have a shorter or longer softening curve (Figure 2b), respectively, and therefore the failure point ($\delta^{f}$) will be computed in a smaller or bigger range, respectively. The result of such assumption has affected the nonlinear part of the stiffness responses to gradually (B2-S-1) or sharply (Be-S-2) be reduced (Figure 7a).

Figure 7b shows the effect of fracture energy variation on the nonlinear part of the stiffness curve, to sharply degrade as a result of smaller energy value, or smoothly degrade as a result of a bigger energy value. Therefore, the composite beam with the assumption of bigger fracture energy fails later due to the larger $\delta^{f}$ value (Figure 2c).

Figure 7c illustrates the effect of both strength and energy values on the stiffness response of the composite beam. Considering the simultaneous variation of these two parameters, a similar value of displacement at failure ($\delta^{f}$) is calculated, as shown in Figure 2d. A proportional variation of these parameters with respect to ref data resulted in a smooth degradation of the stiffness response in the nonlinear section. 

#### 5.2.2. Thin Composite Plate under Three-Point Bending

The load-deflection responses of a thin CFRP composite plate under bending load are presented based on different damage properties, and shown in Figure 8. The results are interpreted with respect to the shapes of the bilinear models that varied based on different property assumptions as shown in Figure 2. It should be noted that, in thin composite plate, the specimen shows a better deformation flexibility before failure. The results are shown for the maximum deflection of 40 mm, where a negative slope in the stiffness response is computed.

Figure 8a shows the effect of variation on the strength property, which is obvious in the P1-S-1 with 50% smaller strength value compare to ref data. However, a larger value of strength (P1-S-2) has not significantly changed the global stiffness response, which is due to the less capacity of the composite structure (thin plate) in generation of stress value to the strength level. It is notable that, the stress distribution is higher at the middle section of the plate where normally shows a bigger deflection; however, this value changes from a location to another. Therefore, in some locations the stress may exceed the strength, however, those locations should be sufficiently big to change the global stiffness response of the structure. This fact is more effective when using smaller strength value, that increased the area of the regions where stress exceeds the strength of the structure, as highlighted in the result of P1-S-1 case. 

Figure 8b indicates the effect of fracture energy variation on the global stiffness response of the thin composite plate. Results indicated no changes in the stiffness response while the fracture energy value is significantly changed. From the damage mechanics point of view, the effect of fracture energy will appear if the damage initiation criteria are satisfied (Equations (2a)–(2d)), then the damage propagation is computed in the simulation process. In addition, the region undergone damage propagation should be large enough to affect the global stiffness response of the structures, which is not applicable in the considered thin plate. Therefore, the reason for the degradation of the stiffness response to a negative value, is due to the structural behavior of the thin plate and its flexibility. As the thin plate is loaded under three-point bending, a bigger area of the plate moved in between the support-span through the plate/roller sliding process (as the deflection getting larger ≈ 30–40 mm). Such a movement increases the effective length of the plate against bending, and results in lowering the global stiffness response. It is worth to note that, this plate is not a good case for research when the objective is the constitutive modeling or damage characterization of the composite materials, as the structure is not capable to reflect the structural response based on the material property variation. 

Figure 8c demonstrates the effect of both strength and energy values on the global stiffness of the thin composite plate. As explained before, the single variation of strength and fracture energy have not been able to change the stiffness response, which is also seen when both parameters are changed. The thin composite plate is introduced as a good example, that should not be used for the damage modeling and characterization of composite materials.

#### 5.2.3. Thick Composite Plate under Three-Point Bending 

The load-deflection responses of a thick CFRP composite plate under three-point bending load based on various damage properties, are presented in Figure 9. The results are interpreted with respect to the shapes of the bilinear model that varied based on different property assumptions as shown in Figure 2. It should be noted that, the reaction force obtained from the thick composite plate is much higher than thin plate (Section 5.2.2). Therefore, it is expected to capture very much different structural responses compare to the previous case, which helps to highlight the objective of this study. It is worth mentioning that a notable higher global stiffness is computed at 4–7 mm deflection in all cases, which is the result of saddle deformation of the composite plate during the bending process [1,17]. A small magnitude of this effect is also seen in the thin plate case (Figure 8, at 11 mm deflection). This effect is normally bigger for specimen with larger width.

Figure 9a indicates a significant influence of the strength variation, which not only changes the maximum reaction force of the structure, but also notably changes the global stiffness (slope of the curve) of the structure (although the elastic properties of all three cases are kept similar to ref data). The deviation of the stiffness value from the ref data is visible at around 500 N force in P2-S-1 case, and around 1000 N for P2-S-2 case. Such deviation is smaller for P2-S-1 case with lower strength value, because that is the point where the stress exceeds from the strength in the middle of the structure which causes the saddle deformation. It should be noted that, the stress exceeding the strength is a sign of damage propagation, that could also have effect on the reduction of the structural stiffness.

Figure 9b shows the effect of fracture energy variation on the global stiffness response of the thick composite plate. It is very interesting to see that, although the strength values are kept similar to ref data in all cases, but the maximum reaction force of the structures has changed for +10% in P2-E-1 case, and −6% in P2-E-2 case compared to ref data. This effect is due to a steeper softening curve and smaller displacement at failure ($\delta^{f}$) for P2-E-1 case, as shown in Figure 2c, which result in the propagation of damage in a bigger area.

Figure 9c illustrates the effect of both strength and energy values on the global stiffness of the thick composite plate. The influence of single damage parameters variation (Figure 9a,b), is visible with slightly higher magnitude. The maximum reaction force and displacement of the P2-SE-2 case are notably higher than P2-S-2. Oppositely, the maximum reaction force and displacement of the P2-SE-1 case are notably lower than P2-S-1.

It is worth to mention that, similar example to the thick composite plate in this study, could be a good case for researches related to a new damage modeling and characterization of FRP laminated composites. Using the thick composite plate, the existence of any inaccuracy in the determination of material properties or damage model could be highlighted, as the property changes result in the variation of the linear and nonlinear response of the composite structure.

#### 5.2.4. Composite Profile under Flexural Loading 

The load–deflection responses of hat-stiffened composite structure under the four-point bending load, based on various damage properties, are presented in Figure 10. The results are construed with respect to various shapes of the bilinear models as shown in Figure 2. Figure 10a demonstrates the effect of the strength variation on the global stiffness response, maximum reaction force, and displacement of the profile structure. Similar to the case of thick composite plate (Figure 9), a notable change in the global stiffness (slope of the linear part of the load–displacement curve) is computed at 8–9 mm deflection in all cases compare to ref data. The deviation of the stiffness value from the ref data is visible at around 400 N force in Pr-S-1 case, which is at around 800 N for Pr-S-2 case. Similar to the case of thick composite plate, the deviation is smaller for Pr-S-1 case as the strength is lower, which causes early stress exceeds of the strength in the mid-section of the structure, that results in lower capacity of the structure to resist the load. It is worth to mention that, the nonlinear portion of the Pr-S-2 is very short (displacement 54–57.5 mm), which is due to the steeper softening curve and smaller value of displacement at failure ($\delta^{f}$), as described in Figure 2b.

Figure 10b indicates the effect of fracture energy variation on the global stiffness response of the composite structure. It is important to highlight that, although the strength values are kept similar in all cases, however, the maximum reaction force of the structure is computed 17% lower in Pr-E-1 case compared to ref data. On the other hand, the nonlinear part of the stiffness response is shown around 300 N higher reaction force compared to ref data, before the final failure of the structure. As mentioned before, this effect is due to a steeper softening curve (and smaller displacement at failure ($\delta^{f}$)) of the ref data compared to Pr-E-2 case, as shown in Figure 2c.

Figure 10c illustrates the effect of both strength and energy values on the global stiffness of the composite structure. The effect of both damage parameters that were individually illustrated in Figure 10a,b, is visible with slightly higher magnitude. Similarly, the maximum reaction force, as well as the displacement range in the nonlinear section (54–77 mm) of the Pr-SE-2 case is very much bigger than Pr-S-2. Notably, the maximum reaction force of the Pr-SE-1 case is lower than Pr-S-1. 

The investigation on this structure provides important data on the dependency of the stiffness response of the structure to the damage model and parameters. This is important as the design of composite structures inevitably depends on the damage investigation and the determination of the inelastic behavior of the structure with respect to the stiffness response.

## 6. Concluding Remarks

This study focused on the dependency of the stiffness response or load-deformation/displacement behavior of CFRP composite materials and structures, to the elastic and damage properties. This is because the stiffness behavior is the most important response that frequently has been utilized for the validation of the theoretical constitutive models, damage theories and mechanical characterization of composites, as well as for the design of composite structures under severe loading. In this regard, the effects of sets of individual/group of mechanical properties including the elastic and shear moduli, strengths, and fracture energies, are studied on the stiffness response of CFRP composite at material and structural levels. These properties are responsible to model different intralaminar failure modes of matrix cracking/crushing, lamina shear failure, and fiber breakage/buckling in composite lamina at meso scale. Four different specimens including a [45]_5_ UD laminated composite beam under tension in material level, two thin and thick CFRP composite plates under three-point bending in structural level, and an aerospace case of hat-stiffened composite structure under flexural loading, were used in a FE environment to apply the elastic-damage model and compute the stiffness response based on different mechanical properties. A set of validated mechanical properties, model and simulation process were used to obtain a reference data, that was utilized to examine different possible scenarios of material and structural behaviors. 

The result indicated a direct dependency of the stiffness response of the composite at material level to the mechanical properties. However, the stiffness behavior of the composite structures depends both on the structural configuration, geometry, lay-ups as well as the mechanical properties of the CFRP composite. The elastic properties directly affect the initial slope and linear response of the composite at material level. However, the composite structures showed two slopes in the linear part of the stiffness curve, in which the initial slope (e.g., Figure 9c, within the deflection values of 0–5 mm) depends to the elastic properties, while the secondary slope (e.g., Figure 9c, within the deflection value of 5 mm to the deflection at maximum load) depends to the geometry of the structure and elastic-damage properties. In this regard, the value of maximum reaction force and displacement of the composite structures are highly dependent not only to the mechanical properties, but also to the geometry and the configuration of the structure. 

Since the stiffness curve is frequently used in the design and analysis of advanced structures, structure cases similar to the case in this studies are introduced as good examples, if the objectives of a research is the investigation of a new theoretical modeling or characterization of FRP composites to highlight the accuracy of material model and parameters (e.g., thick composite plate, Section 5.2.3), or if the objective is the design of composite part and defining the tolerance of the structure by using a mean to the mechanical performance (e.g., hat-stiffened composite structure, Section 5.2.4). 

## Figures and Tables

**Table 1:** Elastic and damage properties of UD CFRP composite lamina.

Elastic Properties				Damage Model Parameters				
Sets 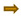	1/2Ref	Ref	3/2Ref			1/2Ref	Ref	3/2Ref
E_11_, GPa	52.75	105.5	158.25	Longitudinal tensile strength, MPa	*X_T_*	670	1340	2010
E_22_, GPa	3.6	7.2	10.8	Longitudinal compressive strength, MPa	*X_C_*	596	1192	1788
E_33_, GPa	3.6	7.2	10.8	Transverse tensile strength, MPa	*Y_T_*	9.8	19.6	29.4
G_12_, GPa	1.7	3.4	5.1	Transverse compressive strength, MPa	*Y_C_*	46.15	92.3	138.45
G_13_, GPa	1.7	3.4	5.1	Longitudinal shear strength, MPa	*S_L_*	25.5	51	76.5
G_23_, GPa	1.26	2.52	3.78	Transverse shear strength, MPa	*S_T_*	11.5	23	34.5
ν_12_	0.34	0.34	0.34	Longitudinal tensile fracture energy, N/mm	*G_XT_*	24.2	48.4	72.6
ν_13_	0.34	0.34	0.34	Longitudinal compressive fracture energy, N/mm	*G_XC_*	30.15	60.3	90.45
ν_23_	0.378	0.378	0.378	Transverse tensile fracture energy, N/mm	*G_YT_*	2.25	4.5	6.75
				Transverse compressive fracture energy, N/mm	*G_YC_*	4.25	8.5	12.75

**Table 2:** List of FE models of CFRP composite materials and structures.

No.	Levels	Composite Case	Specification	Load	Dimension
1	Material	Beam	[45]_5_	Tensile	200 × 20 × 1 mm^3^
2	Structure	ThinPlate 1	[60/45/90/−45/30]	Three-point bending	150 × 60 × 1 mm^3^
		ThickPlate 2	[45_3_/90_3_/0_3_/−45_3_/45_3_]	Three-point bending	150 × 60 × 3 mm^3^
3	Super-structure	Profile	Hat structure: [45/−45/90/45/90] Plate structure: [45/−45/45/−45/45]	Four-point bending	Refer to Figure 3

**Table 3:** Different sets of elastic-damage material properties.

No.	Properties	Cases	Property Code	FE Model	Case Code
1	Elastic Properties (Figure 2a)	**1**	Ep-1	Beam	**Be-Ep-1**
				Plate1	**P1-Ep-1**
				Plate2	**P2-Ep-1**
				Profile	**Pr-Ep-1**
		**Ref**	Ep-Ref	Beam	Be-Ep-Ref *
				Plate1	P1- Ep-Ref **
				Plate2	P2- Ep-Ref ***
				Profile	Pr-Ep-Ref ****
		**2**	Ep-2	Beam	**Be-Ep-2**
				Plate1	**P1-Ep-2**
				Plate2	**P2-Ep-2**
				Profile	**Pr-Ep-2**
2	Strength values (Figure 2b)	**1**	S-1	Beam	**Be-S-1**
				Plate1	**P1-S-1**
				Plate2	**P2-S-1**
				Profile	**Pr-S-1**
		**Ref**	S-Ref	Beam	Be-S-Ref *
				Plate1	P1-S-Ref **
				Plate2	P2-S-Ref ***
				Profile	Pr-S-Ref ****
		**2**	S-2	Beam	**Be-S-2**
				Plate1	**P1-S-2**
				Plate2	**P2-S-2**
				Profile	**Pr-S-2**
3	Energy values (Figure 2c)	**1**	E-1	Beam	**Be-E-1**
				Plate1	**P1-E-1**
				Plate2	**P2-E-1**
				Profile	**Pr-E-1**
		**Ref**	E-Ref	Beam	Be-E-Ref *
				Plate1	P1-E-Ref **
				Plate2	P2-E-Ref ***
				Profile	Pr-E-Ref ****
		**2**	E-2	Beam	**Be-E-2**
				Plate1	**P1-E-2**
				Plate2	**P2-E-2**
				Profile	**Pr-E-2**
4	Strength and Energy values (Figure 2d)	**1**	SE-1	Beam	**Be-SE-1**
				Plate1	**P1-SE-1**
				Plate2	**P2-SE-1**
				Profile	**Pr-SE-1**
		**Ref**	SE-Ref	Beam	Be-SE-Ref *
				Plate1	P1-SE-Ref **
				Plate2	P2-SE-Ref ***
				Profile	Pr-SE-Ref ****
		**2**	SE-2	Beam	**Be-SE-2**
				Plate1	**P1-SE-2**
				Plate2	**P2-SE-2**
				Profile	**Pr-SE-2**

Note; Each set of data presented by *, **, ***, and **** are same. Detail info of the FE models and geometry are provided in Table 2, and Figure 3.

## Data Availability

Not Applicable.
